# Research on the Application and Mechanisms of Electroactive Microorganisms in Toxicants Monitoring: A Review

**DOI:** 10.3390/toxics12030173

**Published:** 2024-02-24

**Authors:** Fei Xing, Liang Duan, Haiya Zhang, Hengliang Zhang, Shilong Li

**Affiliations:** 1 State Key Laboratory of Environmental Criteria and Risk Assessment, Chinese Research Academy of Environmental Sciences, Beijing 100012, China; feixgy@126.com (F.X.); flying850612@126.com (H.Z.); 201831470030@mail.bnu.edu.cn (H.Z.); slone_li@126.com (S.L.); 2 State Environmental Protection Key Laboratory of Estuarine and Coastal Environment, Chinese Research Academy of Environmental Sciences, Beijing 100012, China

**Keywords:** microbial fuel cell (MFC), electroactive microorganisms, toxic monitoring, influence factor, mechanisms

## Abstract

A biological treatment is the core process for removing organic pollutants from industrial wastewater. However, industrial wastewater often contains large amounts of toxic and harmful pollutants, which can inhibit the activity of microorganisms in a treatment system, precipitate the deterioration of effluent quality, and threaten water ecological security from time to time. In most of the existing anaerobic biological treatment processes, toxic effects on microorganisms are determined according to the amounts of end-products of the biochemical reactions, and the evaluation results are relatively lacking. When microorganisms contact toxic substances, changes in biological metabolic activity precede the accumulation of reaction products. As sensitive units, electroactive microorganisms can generate electrical signals, a change in which can directly reflect the toxicity level. The applications of electroactive microorganisms for the toxicity monitoring of wastewater are very promising. Further attention needs to be paid to considering the appropriate evaluation index, the influence of the environment on test results, mechanisms, and other aspects. Therefore, we reviewed the literature regarding the above aspects in order to provide a research foundation for the practical application of electroactive microorganisms in toxicant monitoring.

## 1. Introduction

Microbial fuel cells (MFCs) are reactors that use electroactive microorganisms to generate electricity [1]. In addition to being used in energy production [2,3,4] and wastewater treatments [5,6,7,8], electroactive microorganisms are often used as a sensitive unit for detecting water quality [9,10,11]. Electroactive microorganisms can reflect the magnitude of a toxic effect through changes in an output electrical signal, so they can be applied to the water quality monitoring [12] of domestic sewage [13,14], petrochemical wastewater [15], rivers, and groundwater [11,16], and they can even be used to monitor the damage inflicted by acid rain on crops [17].

The research and applications in the toxic monitoring of wastewater are very promising. Biological treatment is the core process in removing organic pollutants in industrial wastewater [18]. However, industrial wastewater often contains numerous toxic and harmful pollutants, which inhibit the activity of microorganisms in the treatment system [19], precipitating the deterioration of effluent water quality and threatening water ecological safety.

**Figure 1:**
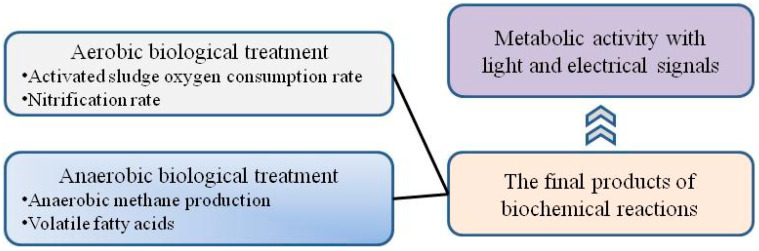
Characteristics of toxicity assessment methods.

Toxicants in wastewater will affect microorganisms in biological treatment systems. According to the type of sewage-focused biological treatment process used, biological treatment can be divided into aerobic biological treatments and anaerobic biological treatments, as shown in Figure 1. The toxicity measurement approaches [20] of aerobic biological processes are relatively mature, and commonly used methods include the activated sludge oxygen consumption rate inhibition test [21], the nitrification rate inhibition test [21], etc. For many high-concentration refractory organic wastewaters, before aerobic biological treatment, anaerobic pretreatment can reduce the concentration of organic matter and improve biodegradability, so the anaerobic biological treatment unit bears the brunt of the impact. Therefore, the evaluation and control of the toxicity of wastewater via anaerobic processes deserve great attention. However, the current domestic and foreign toxicity measurement methods are still limited to the direct determination of the final products of biochemical reactions, and these methods mainly include assessing anaerobic methane production via monitoring the methane content [18], evaluating hydrolytic acidification by monitoring the content of volatile fatty acids [18], and so on. It is well known that, in the process of contact between microorganisms and toxic substances, changes in biological metabolic activity precede the accumulation of reaction products. Therefore, the method of expressing biological metabolic activity with light and electrical signals, thereby indicating the toxicity of wastewater, offers the advantages of fast detection speed and strong sensitivity and is a promising method for assessing the toxicity of wastewater.

## 2. Principle and Application of Electroactive Microorganism in Toxicants Monitoring

### 2.1. The Principle of MFCs

**Figure 2:**
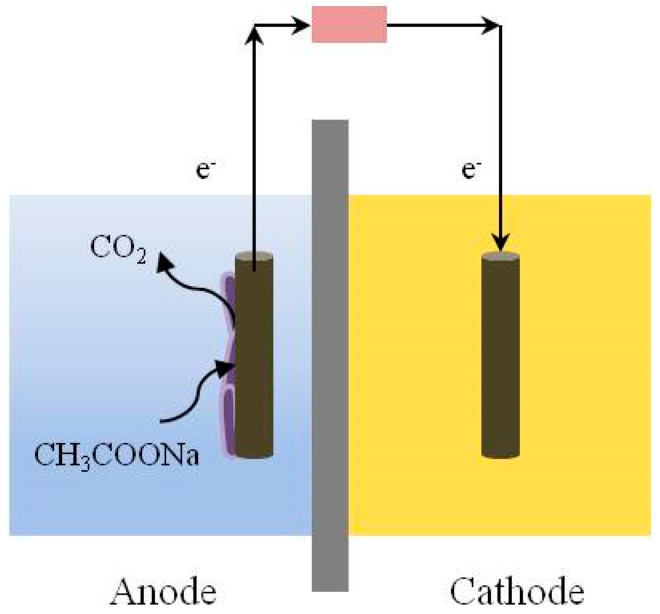
A schematic diagram of the structure of an MFC.

Using microorganisms to efficiently convert chemical energy into electrical energy is the working principle of MFC [22]. A schematic diagram of the structure of an MFC [1] is shown in Figure 2. The electroactive microorganisms at the anode degrade organic matter through biological oxidation reactions and produce electrons, which are transferred to the outside of the cell, and protons are released into the solution at the same time. The electrons are transferred to a cathode via an electrode, an external wire, and load, and the protons that pass through the proton exchange membrane enter the cathode chamber [22]. The electrons, the protons, and the final electron acceptor complete the reduction reaction at the cathode [3].

Electroactive microorganisms can indicate the inhibition of toxicants because these microorganisms are very sensitive to environmental changes. When the influent contains toxicants, the activity of microorganisms is inhibited [23], and the voltage or current generated immediately decrease [24]. Changes can reflect the concentration and the toxicity of the toxic substances.

Electroactive microorganisms mainly originate from soil, river bottom mud [6], deep sea rock mud, and activated sludge from sewage treatment plants [25]. Taxonomic statistics of electroactive microorganisms isolated from different systems have revealed that these microorganisms are mainly distributed in the phyla Proteobacteria, Firmicutes, Acidobacteria, Bacteroidetes, etc. [1,26]. Proteobacteria is an important category of electrically active microorganisms, among which the *Shewanella* spp. and the *Geobacter* spp. are representative model bacteria.

### 2.2. Configuration of MFCs

The configurations of MFCs are mainly dual-chambered and single-chambered. The cathode can be a biological or chemical cathode; in the latter, potassium ferricyanide is often used. The use of a chemical such as Fe (III) can improve the energy output of an MFC, but it also involves chemical losses in the cathode. The classic reactor format of an MFC for inhibitory responses is shown in Figure 2. A reactor with a small volume usually has a lower internal resistance and a higher sensitivity. For example, the micro-MFCs fabricated by Di Lorenzo using 3D-printing technology can rapidly detect cadmium in water at 1–25 μg/L [27]. The cathode-shared MFCs sensor array proposed by Jiang was applied to monitoring toxic substances in reclaimed water [28]. Zhao demonstrated the feasibility of using both bioanodes and biocathodes for suppression detection with continuous flow membrane-less MFCs [7]. Qi combined luminescent bacteria with electroactive microorganisms, allowing the simultaneous detection of electrical and optical signals [29].

The cathode of a single-chamber MFC is often an air cathode [6], but the proton exchange membrane has difficulty in completely isolating the air, and oxygen can therefore infiltrate into the anode, snatching electrons and reducing the sensitivity of the anode. With the increase in operating time, the side of the proton exchange membrane in contact with the air is prone to the formation of a biofilm [25] or salting out [30], increasing resistance. Air cathodes have been modified [31] by means such as the use of nano silver particles [32], quaternary ammonium salt [33], or enrofloxacin [34] to reduce the effects of this phenomenon. The half-wavelength alternating current is applied to counteract fouling and purify air cathodes [31]. Ionic liquids’ proton-exchange membranes have recently received widespread attention [8]. They have unique physicochemical properties (in a liquid state at room temperature), containing organic cations [35] presenting excellent conductivity and thermal stability [36,37], and they can also reduce the size of cathode biofilms.

### 2.3. Electrode Material Modification

Many scholars are committed to the development of new electrode materials by adding active agents or modifying materials to improve the biocompatibility of the electrode. In doing so, more electroactive microorganisms can be enriched on the electrode surface, and the conductivity between the biofilm and the electrode can be improved. The transfer efficiency of MFCs can be improved [38].

The superhydrophilic semiconductor polydopamine is an efficient anode modification material that can shorten the startup time of MFCs and increase power density. The experimental results show that, after adding the polydopamine, the startup time of MFCs is shortened from 88 h to 76 h, the maximum power density is increased from 613 ± 9 to 803 ± 6 mW/m^2^, and the power generation efficiency is increased by 29%. In addition, polydopamine can affect the anode microbial community structure, increasing the proportion of Proteobacteria and Firmicutes [39].

Zhang developed a novel graphene and manganese anode carbon felt coating using graphene and manganese oxide. This binder-free anode material has excellent electrical conductivity and a large surface area, resulting in a 154% increase in maximum power density, with a final value of 2065 mW/m^2^ [38].

### 2.4. Application of Electroactive Microorganisms in Toxicants Monitoring

Kim and colleagues used MFCs for toxic substances detection in 1999. After decades of development, they are now suitable for testing various wastewaters and can enable the toxicity measurement of various substances such as organics, antibiotics, and heavy metals [40]. The common forms of MFC used for toxicity monitoring include single-chamber and double-chamber designs, and the electrical signals monitored include voltage, current, and power. Table 1 shows the application of the electroactive microorganisms reported in a toxicity assessment.

## 3. Common Indicators for the Toxicity Assessment of Electroactive Microorganisms

The inhibition degree of electroactive microorganisms can be compared by response time or inhibition. Response time consists of the time when a current begins to significantly drop [40,52]. The magnitude of the response level can be calculated using current (I) [27], voltage (U) [28,53], and charge [24]. The difference between current and voltage changes can be directly indicated by ΔI and ΔU [52,54,55], and the corresponding calculations are shown in Equations (1) and (2), respectively.

Δ*I* = (*I*_nor_ − *I*_tox_) (1)

Δ*U* = (*U*_nor_ − *U*_tox_) (2)

Among them, *I*_nor_ and *U*_nor_ are stable electrical signals (mA or mV) under normal conditions; *I*_tox_ and *U*_tox_ are the signals (mA or mV) after adding toxic substances; and Δ*I* and Δ*U* are the changes in current and voltage (mA or mV) with toxic substances.

Among the above indicators, the voltage inhibition rate is currently the most commonly used indicator in research for indicating inhibition [28,40,56]. The calculation method is as follows (Equation (3)):

*IR*_U_ (%) = 100 × (*U*_nor_ − *U*_tox_)/*U*_nor_ (3)

Among the terms of the equation above, *IR*_U_ is the voltage inhibition rate (%); *U*_nor_ is the maximum voltage (mV) before adding toxic substances; and *U*_tox_ is the voltage (mV) after adding toxic substances.

There are two ways to calculate the voltage inhibition rate. If the inhibition rate is calculated according to the maximum voltage in one power generation cycle, the results can be obtained after two cycles with and without toxic samples. Therefore, this method takes a long time: for 2~4 mg/L Cu^2+^, the calculation period of the voltage inhibition rate is 50–60 h [53].

**Figure 3:**
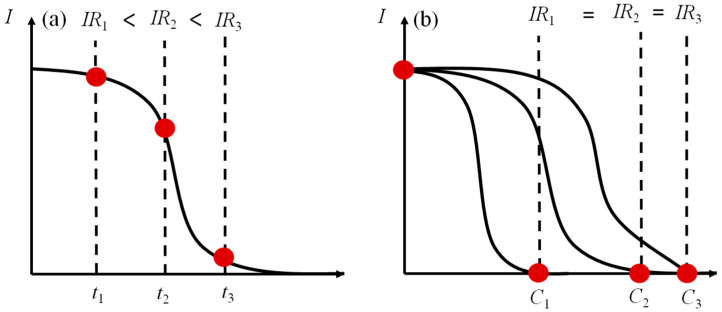
Voltage inhibition rate under different conditions: (**a**) inhibition rate of the same concentration at different times; and (**b**) inhibition rate at different concentrations and times.

Another method consists of calculating the inhibition rate according to the voltage values before and after the addition of toxic samples in one power generation cycle. This method takes a relatively short time. For example, it takes 4 h for a voltage inhibition rate of 5 mg/L of Cu^2+^ to reach 30% [56]. But, as the measurement time is prolonged, the voltage may continue to decrease, so the inhibition rate calculated at different times is different (as shown in Figure 3a). The choice of reaction endpoint varies in studies, ranging from 10 min [27] to several hours [24], making it difficult to compare the levels of toxicity with different studies.

In addition, the electrical signal curve usually changes with different concentrations of a sample, as shown in Figure 3b. The electrical signal immediately decreases with the addition of a higher-concentration toxic sample, and it decreases after a significant hysteresis period at a lower concentration. But, the final stable voltage is almost the same as that at a high concentration.

## 4. Factors Affecting the Toxicity Assessment of Electroactive Microorganisms

The factors influencing the toxicity assessment of electroactive microorganisms are the pH, the temperature, the flow rate, the incubation time, the acetate concentration, and the sodium chloride concentration. Among these, temperature, pH, acetate concentration, and ionic salinity are the characteristics of water quality related to a sample. These factors affect the activity and electrochemical performance of anode biofilms. Electroactive microorganisms are especially sensitive to acids, which affect microbial activity [28]. Temperature is associated with the bacterial metabolism. Chouler found that, when the temperature was changed in the range of 15–35 °C, the output current of MFCs changed by only 8% [57]. Studies have suggested that neutral pH conditions at room temperature are more suitable for the growth of microorganisms [58,59]. Acetate concentration and sodium chloride concentration will affect the components content of extracellular polymers in microorganisms and indirectly affect the performance of the bioanode [60]. The flow rate and the incubation time are influencing factors related to operating conditions, and the flow rate affects biofilm formation. Different thicknesses of the biofilms formed on the electrode surface over different incubation times directly affect the response time. It has been found that, when the concentration of sodium acetate is 1 g/L and the corresponding biofilm is cultured for about 7 days, the monitoring sensitivity of toxic substances is higher. Other monitoring conditions need to be scrutinized to improve the monitoring accuracy of sensors.

### 4.1. Flow Rate

Studies have shown that the flow rate affects the power generation and sensitivity of electroactive microorganisms. Di Lorenzo found that reducing the flow rate led to better power generation efficiencies [61]. Shen found that reducing the flow rate could accelerate the response to Cu^2+^, and intermittent nitrogen perturbation could increase the contact speed of toxic substances with microorganisms and accelerate the response to toxic substances [56].

Chen found that higher flow rates favored matrix diffusion into biofilms but increased biofilm density, causing the rate of matrix diffusion within the biofilm to decrease [62]. Under a high flow rate, a biofilm is dense with a high shear rate, and toxic substances do not easily diffuse into the biofilm, a circumstance which is not conducive to the system’s response to said toxic substances [56,62]. A high flow rate causes an irreversible loss of biofilm, so a reasonable shear rate has an important impact on the attachment of electroactive microorganisms [63]. The effects of flux on biofilm properties such as biofilm density and porosity have been reported. It is necessary to carry out research on the responses of different flow rates and explore reasonable flow rates suitable for responses.

### 4.2. Culture Time

One of the advantages of MFCs is that they can operate for a long time and generate energy continuously [64], but long-term operation leads to the formation of thick biofilms [56]. A thick biofilm reduces the performance of an anode. The electron transfer of biofilms depends on the conductivity of the substrate, and the thicker the biofilm, the lower the conductivity [65]. As the thickness of the biofilm increases, the resistance to biomass production and mass transfer increases [56]. After long-term operation, a thick biofilm reduces the electron transfer efficiency and increases charge transfer resistance [66,67,68]. The material exchange rate can be accelerated by scraping the biofilm to improve the performance of the anode [69]. Studies have shown that long-term culturing will reduce anode performance and is unfavorable in terms of responses to toxic substances. It is necessary to study responses to culture time in order to improve the sensitivity of the responses of MFCs by enhancing anode redox capacity and microbial community structure.

### 4.3. Substrate Concentration

The available substrates for electroactive microorganisms are acetate, ethanol, glucose, etc. Substrate oxidation involves many electrochemical and biochemical reactions, and the generated current can indicate the oxidation rate of the substrate [40]. Different substrates of MFCs have different electricity production capacities and byproducts. When the substrate is glucose, glucose is hydrolyzed to form acetone and then hydrolyzed to lactic acid and acetic acid. There are three main stages in generating electrons [70]. Compared with low-molecular-weight substrates, glucose is less efficient in electricity production [71], and electroactive microorganisms are more likely to utilize low-molecular-weight substrates (such as acetate) as electron sources [71,72] and respond faster.

Ledezma studied the effect of substrate concentration on the electricity production of MFCs and found that, when the acetate concentration was greater than 100 mM, the current was no longer enhanced, and the substrate reached a saturated concentration, which was in line with the growth in microbial-saturated substrates’ kinetics [73]. Chouler added 0.1–200 mM of potassium acetate, and the response to potassium acetate conformed to the Mono equation [57]. For marine microbial fuel cells cultured with mixed bacteria, current density is close to a constant value when the acetate concentration is greater than 0.50 mM [74].

Earlier studies showed that the current in response to substrate concentration corresponds to a first-order equation: that is, a finite substrate concentration corresponds to a low current density [75]. Acetate concentration is one of the key factors affecting microbial community composition and extracellular polymer composition [76]. There have been many reports on the effect of acetate concentration on electricity production but few on the response to toxic substances. In most studies, the acetate concentration was 1 g/L [77]. Therefore, it is necessary to carry out research on the responses to toxic substances measured for different acetate concentrations in order to obtain relatively accurate response results.

### 4.4. Sodium Chloride Concentration

NaCl concentration affects the activity of anode microorganisms and the electrochemical performance of MFC biosensors [57]. The pH of a solution and that of an electrode are often controlled using a buffer solution [78]. A phosphate-buffered solution is often used for MFCs because its pKa is close to neutral, and its biocompatibility is better [79,80]. The concentration of sodium chloride affects extracellular electron transfer and, thus, the power density of MFCs. Studies have shown that power density and output voltage reach a maximum when the concentration of sodium chloride is 1% (*w*/*v*) [81]. Although increasing the concentration of NaCl can increase the conductivity of the solution, it does not improve the electrical performance [82,83]. When the sodium chloride concentration is 0.1 M, the power density of the *Geobacter* spp. largely fluctuates [84]. Applying more than 0.1 M NaCl changes the bacterial species’ presence in anode biofilms and, ultimately, reduces electricity generation [85]. The effect of sodium chloride concentration on electricity production in MFCs has been reported, but there are few reports on the corresponding response, and the effects on biological activity and redox capacity are even less reported. Therefore, it is necessary to carry out research on different sodium chloride concentrations to ensure reliability.

## 5. Reasons for Indicating the Toxicity of Electroactive Microorganisms

### 5.1. Electron Transfer of Electroactive Microorganisms

Electroactive microorganisms are mostly Gram-negative bacilli, which can oxidize electron donors in the cytoplasm, generate electrons, H^+^, etc., and transfer electrons to electron acceptors through the outer membrane of cells. The generation and transmission of electrons are mainly realized through respiration in the cell and via the nanowires of cytochrome *c* and mediators outside the cell. Respiration occurs in the cell membrane (including the outer membrane, inner membrane, and periplasm). The proteins required for the transfer of electrons are usually five intermediate proteins: reduced coenzyme I, dehydrogenase, ubiquinone, coenzyme Q, and cytochrome [86].

For some specific bacterial strains, such as the *Shewanella* spp. and the *Geobacter* spp. direct electron transfer is considered an efficient electron transfer pathway. Research on the conduction mechanism of nanowires or cytochrome *c* in direct electron transfer can be realized by technical means such as gene chips and gene knockouts. Islam reported that nanowires did not show high electrical conductivity [67]. Through genomics studies, it was found that the electron transfer process of *Shewanella oneidensis* MR-1 involves six cytochromes *c*. The CymA of the inner membrane transfers electrons to Fcc3 and STC in the periplasm and then to the complex protein on the outer membrane. The electron transport process of the outer membrane involves MtrA, MtrB, and MtrC [11,87]. In one study, when the gene related to CymA was knocked out, the rate of metabolism using extracellular solid and dissolved electron acceptors was inhibited [88].

**Figure 4:**
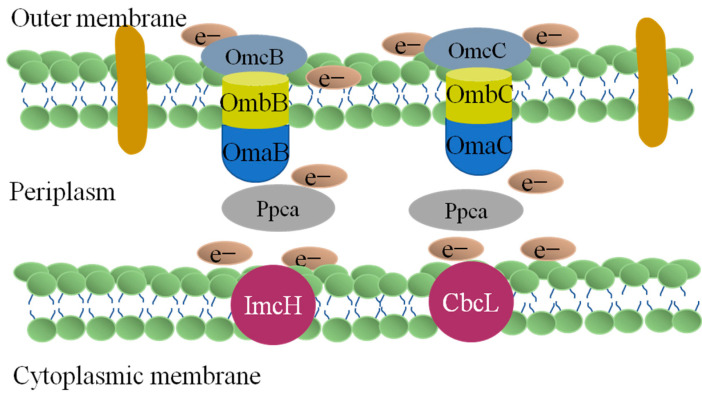
Electron transfer pathways of *Geobacter sulfurreducens* PCA.

The electron transfer process of *Geobacter sulfurreducens* PCA (Figure 4) is more complicated than that of *Shewanella oneidensis* MR-1. Through genomics studies, it was found that lmcH and CbcL in the inner membrane transfer electrons to PpcA in the periplasm; these electrons are then transferred to the four complex proteins on the outer membrane, respectively. The electron transport process of these four outer membrane complex proteins involves 2643, 2644, and 2642; OmaB, OmbB, and OmcB; OmaC, OmbC, and OmcC; and 2725, 2726, and 2742 [11,87].

Electron mediators are redox-active substances that can be used as electron carriers for periodic cyclic electron transfer between extracellular electron acceptors/donors and microbial cells. The indirect extracellular electron transfer mediated by electron mediators enables substances to undergo redox reactions without entering the intracellular membrane and periplasmic space. Electronic mediators include cellular secretions and agents added via exogenous dosing. Among these, the mediators secreted by cells include riboflavin [68], pyocyanin [89], phenols, proteins, and quinines, as well as the exogenous addition of phenazine and neutral red and so on.

### 5.2. Electron Transfer and Metabolism Change between Electroactive Microorganisms

According to the electron transfer mechanism of electroactive microorganisms, the electroactive microorganisms in the anode chamber of MFCs not only use themselves as electron donors and electrode materials as electron acceptors but also complete the transfer and transformation of electrons through other forms. Electroactive microorganisms can cooperate with other microorganisms in a heterotrophic metabolic pattern. Interspecific electron transfer is another syntrophic metabolic mechanism different from intraspecific electron transfer. Microorganisms transfer the electrons generated via the metabolism to other microorganisms through their own nanowires or cytochromes *c* and conductive substances. One study found that *Arcobacter* [90] and *Desulfovibrio* [91] can transfer electrons to each other.

The syntrophic metabolizing microorganisms that transfer energy through interspecific electron transfer channels are Gram-positive bacilli such as *Clostridium pasteurianum*, *Geobacter sulfurreducens,* and anaerobic photosynthetic bacteria. In the system in which *Geobacter sulfurreducens* coexists with other flora, *Geobacter sulfurreducens* oxidizes acetate, and electron transfer is realized by cytochrome *c* and nanowires (Equation (4)) [70,92]. In addition, *Klebsiella*, *Aeromonas,* and *Tolumonas* can carry out direct interspecies electron transfer by oxidizing electrons from glucose through cytochrome *c* to the anode complex (Equation (5)). Therefore, electron transport in a wide variety of mixed bacteria is the result of the coexistence of multiple electron transport modes [70].

(4) $$\mathrm{Acetate}+2H_{2}O\overset{\mathrm{cytochromes}/\mathrm{pili}}{\to }2CO_{2}+8H^{+}+8e^{-}$$

(5) $$\mathrm{NADH}\overset{\mathrm{cytochromes}}{\to }{\mathrm{NAD}}^{+}+H^{+}+2e^{-}$$

Life activities are inseparable from energy metabolism, which directly affects microbial respiration, in turn affecting metabolic flux and redox balance [93]. Organic molecules undergo glycolysis to produce acetyl-CoA and then participate in the tricarboxylic acid cycle. Microbial redox reactions and energy production are inseparable from NAD(H) [94], as NAD(H) is an important carrier for extracellular electron transfer [94,95]. During substrate metabolism, dehydrogenase is an important enzyme involved in redox reactions [96] and can also transfer electrons between metabolic intermediates [97]. Studies have shown that extracellular acetic acid enters the cell and is then converted into acetyl-CoA. After isotope labeling, the enzyme generates ethanol through NADPH. Through metabolomic analysis, it was found that microbial respiration in this context was enhanced, the ratio of ATP/ADP was higher, and the ratios of NAD^+^/NADH and NADP^+^/NADPH were lower [93]. Toxic substances not only inhibit the energy synthesis of microorganisms but also inhibit the enzymes that the microorganisms need to synthesize during respiration [53], blocking the transfer of electrons and causing a voltage drop.

### 5.3. Mechanism of Electroactive Microorganisms to Resist Adverse Environment

i. Changes in the intracellular antioxidant enzymes of electroactive microorganisms

In recent years, scholars have found that membrane damage caused by oxidative stress is the main bactericidal mechanism behind the toxicity of toxic substances to microorganisms [98]. Microbial cell membranes are prone to change after being attacked by pollutants [99], and heavy-metal stress causes microorganisms to produce superoxide radicals or hydrogen peroxide, which damage proteins, lipids, and nucleic acids [100]. Microorganisms secrete various antioxidant enzymes such as superoxide dismutase [101], catalase [101], and glutathione peroxidase to carry out oxidative stress detoxification, thereby protecting cells from oxidative stress damage [102]. Lipids are the main targets of oxidative stress damage. Free radicals directly react with polyunsaturated fatty acids on cell membranes, causing lipid peroxidation, resulting in decreases in cell membrane fluidity, changes in cell membrane properties, and the destruction of cell membrane proteins [103]. The protein aggregation of cell membrane and the loss of activity forms ion channels. Then, the integrity of the cell membrane is destroyed, and the membrane’s permeability is changed [103], finally leading to the disintegration and death of the bacteria. At present, there are relatively few studies on the inhibitory mechanisms of electroactive microorganisms. Therefore, by examining the single-electron transfer pathway related to respiration on the cell membrane and the changes in the related enzymes, it is possible to explore the effects of toxic substance inhibition mechanisms.

ii. Changes in electroactive microbial extracellular polymers

Electroactive microorganisms first contact toxic substances via the extracellular polymer (extracellular polymeric substances, EPSs) on the outermost layer of bacteria. The main components of the extracellular polymer include exopolysaccharide, protein, nucleic acid, and other substances [104]. Regarding the cause of toxicity, it is inseparable from microbial cell membrane adsorption [99]. EPSs are key structures in bacterial cells [105] and have important physiological effects on bacteria [106,107]. The content of EPSs affects the sensitivity of electroactive microorganisms [11,76].

## 6. Toxicity Evaluation Methods of Anaerobic Biological Treatments

For industrial wastewaters with a high organic concentration and a high toxicity [108], hydrolytic acidification is used as the first biological treatment process to improve biodegradability. Therefore, it is necessary to pay attention to the inhibition of hydrolytic acidification and acid production. In addition, due to its short monitoring time, high sensitivity, and automation, the acute toxicity test for luminescent bacteria is often used in various biological toxicity or inhibition tests.

### 6.1. The Toxicity Assay of Anaerobic Methane Production

**Figure 5:**
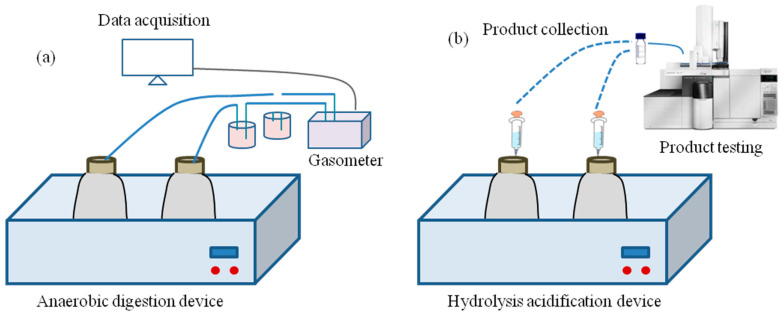
Toxicity evaluation methods: (**a**) the toxicity assay of methane production; and (**b**) the volatile fatty acids assay.

The anaerobic toxicity assay (ATA) [18] based on methane production is a toxicity evaluation method for evaluating the effect of substrates on methanogenesis. Similar to the CO_2_ emission test, the ATA (Figure 5a) measures methane production in batches and uses the ratio of methane production relative to the control to assess substrate toxicity. The ATA method based on methane production is widely used and well developed. In tests conducted in different laboratories, the inhibition rate of 32 mg/L-510 of mg/L 3,5-DCP for microorganisms in an anaerobic treatment system was 50%, as per the evaluation standard [109]. Most ATA experiments are carried out under anaerobic conditions at mesophilic temperatures (about 35 °C). The commonly used calculation method in the ATA consists in determining the specific methane production rate of toxic substances in a sample. The calculation formula is as follows:

*I*_ATA_ = 100% × (1 − *R*_t_/*R*_0_) (6)

Among the terms of the equation above, *I*_ATA_ is the inhibition rate of methane production (%); *R*_t_ is the methane production rate of the tested wastewater group (mL/h); and *R*_0_ is the methane production rate of the blank control group (mL/h).

### 6.2. The Inhibition of Acid Production by Hydrolysis and Acidification

Volatile fatty acids (VFA) are metabolic intermediates that exist in biological reaction systems and can reflect the acid production capacity of fermentation systems [18]. In an anaerobic reactor, the accumulation of VFA (Figure 5b) can reflect the inactive state of hydrolytic acidifying bacteria [19]. Higher VFA concentrations have an inhibitory effect on methanogens. The toxicity of toxic substances can be investigated by comparing the changes in the production rate of VFA before and after adding toxic substances. The formula for calculating the inhibition rate of volatile fatty acid production is as follows:

*I*_VFA_ = 100% × (1 − *R*_t_/*R*_0_) (7)

Among the terms of the equation, *I*_VFA_ is the acid production inhibition rate (%); *R*_t_ is the production rate of total volatile fatty acids (mg/(L·h)); and *R*_0_ is the production rate of total volatile fatty acids in the control group (mg/(L·h))

### 6.3. Evaluation of Relative Luminescence Inhibition

The toxicity evaluation of luminescent bacteria involves the use of photoelectric detection technology, which is applied in the evaluation of pollutants and environmental monitoring. At present, this method has been applied to assess the quality of industrial wastewater and the toxicity of marine sediments and in the toxicity evaluation and environmental monitoring of some toxic organic pollutants and heavy metals.

There are generally three types of expressions for acquiring toxicity evaluation results using luminescent bacteria: the relative luminescence rate, the relative inhibition rate, and *EC*_50_. *EC*_50_ is the concentration of a substance at which the relative light inhibition rate reaches 50% or more. The precision standard [110] stipulates that the relative deviation of the three repeated determination results of a sample should not be greater than 15%.

The toxicity toward luminescent bacteria is expressed by the relative photo inhibition rate, and the calculation formula is as follows:

*I* = 100% × (1 − *R*_t_/*R*_0_) (8)

Among the terms of the equation, *I* is the photoacid production rate inhibition rate (%); *R*_t_ is the light intensity of the sample (cd); and *R*_0_ is the light intensity of the control group (cd).

### 6.4. The Toxicity Assay of Electroactive Microorganisms

Traditional toxicity measurement methods can be used to monitor the types and concentrations of toxic substances in water, but the pre-treatment process is cumbersome, and the monitoring results are often lacking. Compared with traditional analytical methods, electroactive microorganisms do not require complex pre-treatment, are inexpensive, and can be used to effectively monitor toxic and harmful substances, revealing promising application prospects.

When the voltage is maintained at 620 ± 20 mV and kept stable for three cycles, it can be used for toxicity testing. The calculations for the inhibition rate, a toxicity evaluation indicator, include the entire toxic process [45]. The average current inhibition rate refers to the average current, taking into account the stable current of MFCs after adding toxic substances and comparing it with the normal current without toxic substances added.

The average current and its inhibition rate are calculated as follows:

(9) $$\bar{I}=(\int_{t_{1}}^{t_{2}}Idt)/(t_{2}-t_{1})$$

(10) $$I_{I}\;(\%)=100\;\times \;(I_{\mathrm{nor}}-\bar{I})/I_{\mathrm{nor}}$$

Among the terms of the equation above, *t*_1_ is the time it takes for the current to decrease more than 5% of the normal value; *t*_2_ is the time required to stabilize again with a fluctuation of no more than 5%; $\bar{I}$ is the average current of the toxic test; and *I*_nor_ is the normal value before adding toxic substances.

Among all the microorganisms that act as sensing elements, electroactive bacteria and luminescent bacteria are the two most important, because, without any additional chemical mediators, they can generate detectable fluorescence and current as warning signals [11]. Luminescent bacteria have been used in toxicity assays, and TOXcontrol^®^ has developed an online bioluminescence assay system. However, it has been found that, as an indirect measure, luminescent bacteria may be too sensitive and often trigger false positive signals in wastewater assessments [18].

By investigating the toxicity evaluation method, an evaluation method consisting of electrical signals that can output toxicity levels online and in a timely fashion was developed. Electroactive microorganisms have been introduced into wastewater toxicity evaluation as a supplementary method to enrich toxicity evaluation methods and provide technical support for ensuring stable operations in wastewater biological treatment.

## 7. Conclusions and Perspectives

When using indicators to evaluate toxicity levels, the instantaneous values of electrical signals can easily lead to biased results. Before the voltage becomes stable, the inhibition rates at different times inevitably increase over time. Therefore, the selection of evaluation indicators requires consideration of the overall evaluation results. The environment has a significant impact on toxicant monitoring, especially in terms of cultivation time, substrate flow rate, sodium chloride concentration, and acetate concentration, which affect the characteristics and community composition of microorganisms. Therefore, these aspects greatly affect the power generation performance and sensitivity of electroactive microorganisms. In order to apply electroactive microorganisms in wastewater toxicity assessment, it is necessary to clarify the influencing factors and optimal operating conditions. The contributions of electroactive microorganisms in electron generation and transfer are different, and the competitive or complementary effects with respect to toxic substances are not yet clear. It is necessary to reveal their mechanisms with regard to toxic substances based on the characteristics of electron conduction, growth metabolism, and self-defense behavior.

## Figures and Tables

**Table 1:** Application of electroactive microorganisms in the evaluation of toxicants.

Toxicants		Reactor	Signal	Detection Concentration (mg/L)	References
Organics	Formaldehyde	double-chamber	current	0.1% *v/v*	[41]
	Acetic Acid	double-chamber	voltage	15	[42]
	p-Nitrophenol	single-chamber	current	50	[43]
	Azide	single-chamber	current	0.02	[44]
	2,4-Dichlorophenol	double-chamber	voltage	0.7	[45]
	Pyridine	double-chamber	voltage	0.1	[45]
Antibiotics	Levofloxacin	single-chamber	current	0.0001	[46]
	Imipenem	double-chamber	voltage	1.25	[47]
	Tobramycin	single-chamber	current	0.1	[48]
	Neomycin Sulphate	single-chamber	voltage	20	[49]
Heavy metals	Cu(Ⅱ)	double-chamber	current	2	[28]
	Cd(Ⅱ)	double-chamber	current	0.001	[27]
	Cr(Ⅵ)	single-chamber	voltage	1	[50]
	Fe(Ⅲ)	single-chamber	power	2.8	[51]

## Data Availability

No new data were created or analyzed in this study. Data sharing is not applicable to this article.
